# *Gubenyiliu* II Inhibits Breast Tumor Growth and Metastasis Associated with Decreased Heparanase Expression and Phosphorylation of ERK and AKT Pathways

**DOI:** 10.3390/molecules22050787

**Published:** 2017-05-15

**Authors:** Yi Zhang, Gan-Lin Zhang, Xu Sun, Ke-Xin Cao, Ya-Wen Shang, Mu-Xin Gong, Cong Ma, Nan Nan, Jin-Ping Li, Ming-Wei Yu, Guo-Wang Yang, Xiao-Min Wang

**Affiliations:** 1 Department of Oncology, Beijing Hospital of Traditional Chinese Medicine, Capital Medical University, Beijing 100010, China; cmdzhangyi@126.com (Y.Z.); jiayousx@yeah.net (X.S.); kexin309@aliyun.com (K.-X.C.); macong0716@sina.com (C.M.); kkwp263@163.com (N.N.); jin-ping.li@imbim.uu.se (J.-P.L.); yumingwei1120@163.com (M.-W.Y.); guowangyang@sina.com (G.-W.Y.); 2 School of Traditional Chinese Medicine, Capital Medical University, Beijing 100069, China; zhiqiang631015@126.com (Y.-W.S.); gongmuxin@126.com (M.-X.G.); 3 Department of Medical Biochemistry and Microbiology, Biomedical Center, Uppsala University, Uppsala 75123, Sweden

**Keywords:** *Gubenyiliu* II, breast tumor, heparanase, growth factors, ERK and AKT pathways

## Abstract

*Gubenyiliu* II (GYII), a Traditional Chinese Medicine (TCM) formula used in our hospital, has shown beneficial effects in cancer patients. In this study, we investigated the molecular mechanisms underlying the beneficial effects of GYII on murine breast cancer models. GYII showed significant inhibitory effects on tumor growth and metastasis in the murine breast cancer model. Additionally, GYII suppressed the proliferation of 4T1 and MCF-7 cells in a dose-dependent manner. A better inhibitory effect on 4T1 cell proliferation and migration was found in the decomposed recipes (DR) of GYII. Moreover, heparanase expression and the degree of angiogenesis were reduced in tumor tissues. Western blot analysis showed decreased expression of heparanase and growth factors in the cells treated with GYII and its decomposed recipes (DR2 and DR3), and thereby a reduction in the phosphorylation of extracellular signal-regulated kinase (ERK) and serine-threonine kinase (AKT). These results suggest that GYII exerts anti-tumor growth and anti-metastatic effects in the murine breast cancer model. The anti-tumor activity of GYII and its decomposed recipes is, at least partly, associated with decreased heparanase and growth factor expression, which subsequently suppressed the activation of the ERK and AKT pathways.

## 1. Introduction

Breast cancer is one of the most common malignant tumors among women, and its incidence is steadily increasing globally [1]. Although surgical elimination of a tumor is an effective treatment, some patients must undergo chemotherapy or radiotherapy to prevent metastasis. Almost all patients undergoing chemo- or radiotherapy suffer from the toxic effects of treatment, and many patients stop treatment due to severe side effects. To counteract the adverse effects of chemo- or radiotherapy and increase patient tolerance and wellbeing, Traditional Chinese Medicine (TCM), often in the form of a mixture of herbs prepared as a formula, has been applied to patients undergoing chemo- or radiotherapy. Accumulated clinical observations have demonstrated that herbal medicines can greatly improve patient tolerance for several anti-cancer drugs [2,3]. Moreover, some herbs possess anti-cancer properties [4,5,6].

The *Gubenyiliu* II formula (GYII) is a mixture of 13 different species of herbs that was developed at our hospital based on *yiqi* (replenish/positive energy), *huoxuehuayu* (stimulation of blood circulation), and *jiedusanjie* (neutralizing tumor-induced toxicity and tumor resolving) theory. GYII has been administered to diverse cancer patients for more than 30 years and has demonstrated synergetic activity with chemo- and radiotherapy in cancer patients [4,7]. Treating breast cancer patients with Navelbine^®^ and cisplatin combined with GYII achieved better short-term and long-term efficacy, as evaluated by quality of life and carcino-embryonic antigen (CEA) levels [8]. Furthermore, GYII inhibited tumor growth in mice bearing Lewis lung cancer xenografts and induced apoptosis [9]. Following the theory of TCM, GYII was divided into three decomposed recipes (DR); DR1 was prepared from herbs with a *yiqi* effect, while DR2 was prepared from herbs with a *huoxuehuayu* effect, and DR3 was prepared from herbs with a *jiedusanjie* effect. In our previous study, the water-soluble extracts of GYII and its DR2 (which was named as blood activation prescription (BAP)) exerted their inhibitory effects on the growth of MCF-7 human breast cancer xenografts by inducing tumor apoptosis and autophagy [10]. These clinical and preclinical findings led us to further investigate the anti-tumor mechanisms and effective components of GYII in breast cancer. Classical TCM theory is characterized by holism and syndrome differentiation. In cancer treatment, we emphasize the overall regulation of the condition of the body rather than the tumor itself [2]. This kind of concept is similar to Stephen Paget’s seed and soil theory and extensively accepted by cancer researchers [11]. Therefore, the effects of herbs under the guidance of TCM theory can be partly ascribed to affect the microenvironment of tumors [12,13,14], e.g., the extracellular matrix (ECM). The integrity of the ECM plays a critical role in tumor growth and metastasis. Moreover, heparanase, an endo-β-glucuronidase capable of cleaving heparan sulfate (HS), participates in the degradation of the ECM and hence prompts tumor progression and metastasis [15,16]. According to the meta-analysis and the cancer genome atlas (TCGA) data, we found that heparanase expression was up-regulated in most breast cancer specimens, and elevated heparanase expression was associated with increased lymph node metastasis, larger tumor size, higher histological grade, and poor survival, and suggested that targeting heparanase might improve treatments for breast cancer patients [17]. So, it is of sufficient interest to explore the effect of GYII from the perspective of heparanase.

To obtain insights into the mechanisms underlying the effects of GYII and its decomposed recipes, we used in vivo and in vitro models to assess the effect on tumor growth/metastasis and tumor cell proliferation/migration. The effects of the herbal extracts were evaluated in comparison with fucoidan, which is a fucose-containing sulfated polysaccharide derived from brown seaweeds and has been regarded as a potential therapeutic agent for cancers [18]. A previous study showed that fucoidan inhibited 4T1 murine breast cancer growth and metastasis, involving decreased tumor angiogenesis, downregulation of the growth factor’s expression, and phosphorylation of extracellular signal-regulated kinase (ERK) pathways [19]. Our results reveal that GYII and its decomposed recipes exert anti-tumor and anti-metastatic effects in murine breast cancer and the effects are, at least partially, associated with decreased heparanase and growth factor expression, which subsequently suppressed the activation of ERK and serine-threonine kinase (AKT) pathways.

## 2. Results

### 2.1. GYII Inhibited Primary Tumor Growth and Lung Metastasis in a Murine Mammary Carcinoma Model

**Figure 1:**
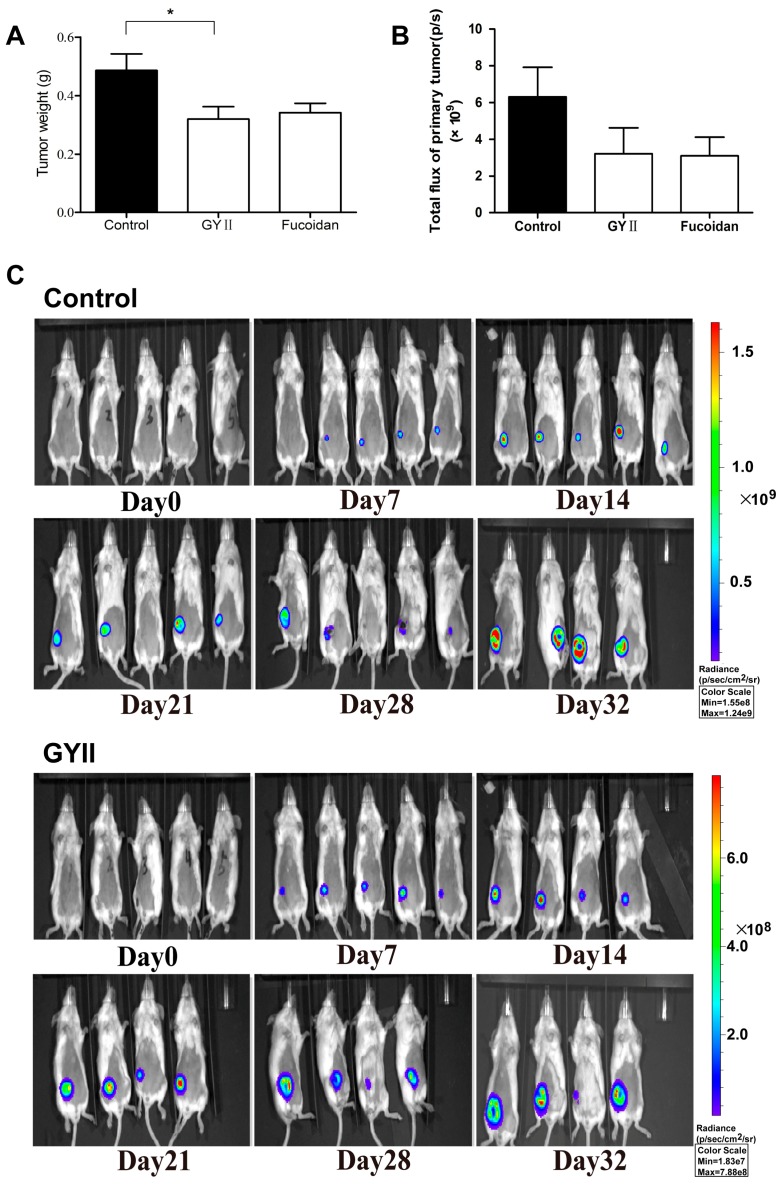
*Gubenyiliu* II (GYII) inhibited tumor growth. (**A**) In a 4T1-luc2 mouse model, the animals were treated daily with the polysaccharide of GYII, fucoidan, or vehicle for 25 days. Tumor weights were reduced in the GYII group (IR = 34%), higher than that of fucoidan (IR = 30%); (**B**) Bioluminescent signals of primary tumors were expressed by total flux and quantitated by photons/second on day 32; (**C**) Representative images of mice in control and GYII groups. Statistical comparisons were performed using one-way analysis of variance (* *p* < 0.05).

**Figure 2:**
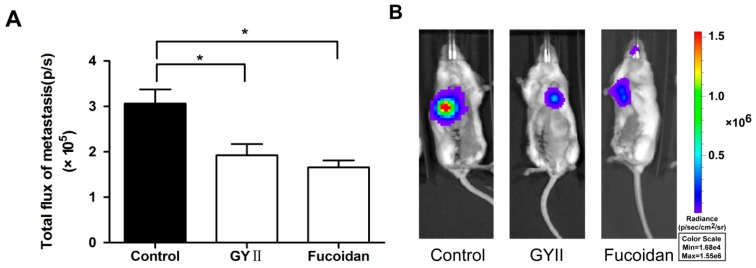
GYII reduced lung metastasis. (**A**) The micro-metastatic bioluminescent signals were detected after resection of the 4T1-luc2 primary tumor on day 32. GYII significantly suppressed lung metastases (* *p* < 0.05); (**B**) Representative bioluminescent images of mice with or without treatment.

The orthotopic 4T1 model was used to compare the effects of GYII and fucoidan on breast tumor growth. In the mammary orthotopic 4T1-luc2 model, primary tumors were detected by both weight and bioluminescent signal. At the test dose, GYII (IR = 34%) showed inhibitory efficacy by weight (Figure 1A). Although there was no significant difference, the mean total flux of the tumor was also decreased after treatment with GYII (3.22 × 10^9^ p/s) and fucoidan (3.11 × 10^9^ p/s) compared with the control group (6.32 × 10^9^ p/s) (Figure 1B). At the end of the study (day 32), mice were examined for evidence of spontaneous metastasis to the lung. Results showed that GYII and fucoidan significantly suppressed the lung metastases by 37.1% and 45.9%, respectively (Figure 2).

### 2.2. Selective Inhibition of Breast Cancer Cell Proliferation by GYII

**Figure 3:**
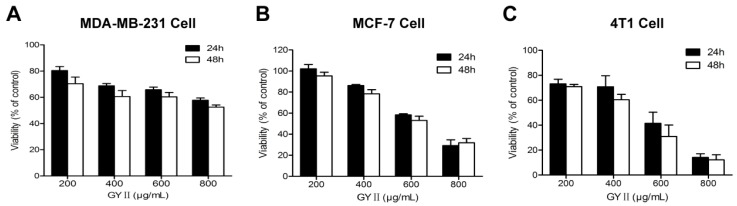
Dose-dependent inhibitory effect of GYII on breast tumor cell proliferation. Three breast tumor cell lines were treated with GYII at different concentrations and cell survival was monitored using the 3-(4,5-dimethyl-thiazol-2-yl)-2,5-Diphenyl tetrazolium bromide (MTT) assay. Absorbance (570 nm) was measured as an indicator of cell survival, and the control group viability was defined as 100%. Proliferation of 4T1 (**C**) and MCF-7 (**B**) cells was dose-dependently inhibited by GYII, while proliferation of MDA-MB-231 cells (**A**) was moderately affected.

Next, we performed a series of in vitro experiments to investigate the effect of GYII on cell models. First, the 3-(4,5-dimethyl-thiazol-2-yl)-2,5-Diphenyl tetrazolium bromide (MTT) assay was used to evaluate the viability of MDA-MB-231, MCF-7, and 4T1 breast cancer cells. The cells were cultured in the presence of GYII at concentrations of 200–800 μg/mL for 24 and 48 h, and a dose-dependent inhibitory effect was produced in both MCF-7 and 4T1 cells (Figure 3B,C). The inhibitory effect was rapid and sustained, as there was no significant difference between the results obtained after 24 and 48 h. In comparison, the inhibitory effect of GYII on MDA-MB-231 cells was moderate (Figure 3A). Because the 4T1 cells appeared to be more sensitive to GYII than the MCF-7 cells (half-inhibitory concentration (IC_50_) of 4T1 cells = 396 μg/mL; IC_50_ of MCF-7 cells = 639 μg/mL), they were used for subsequent studies. 

### 2.3. Differential Inhibitory Effects of GYII and Its Decomposed Recipes on 4T1 Cell Proliferation and Migration

**Figure 4:**
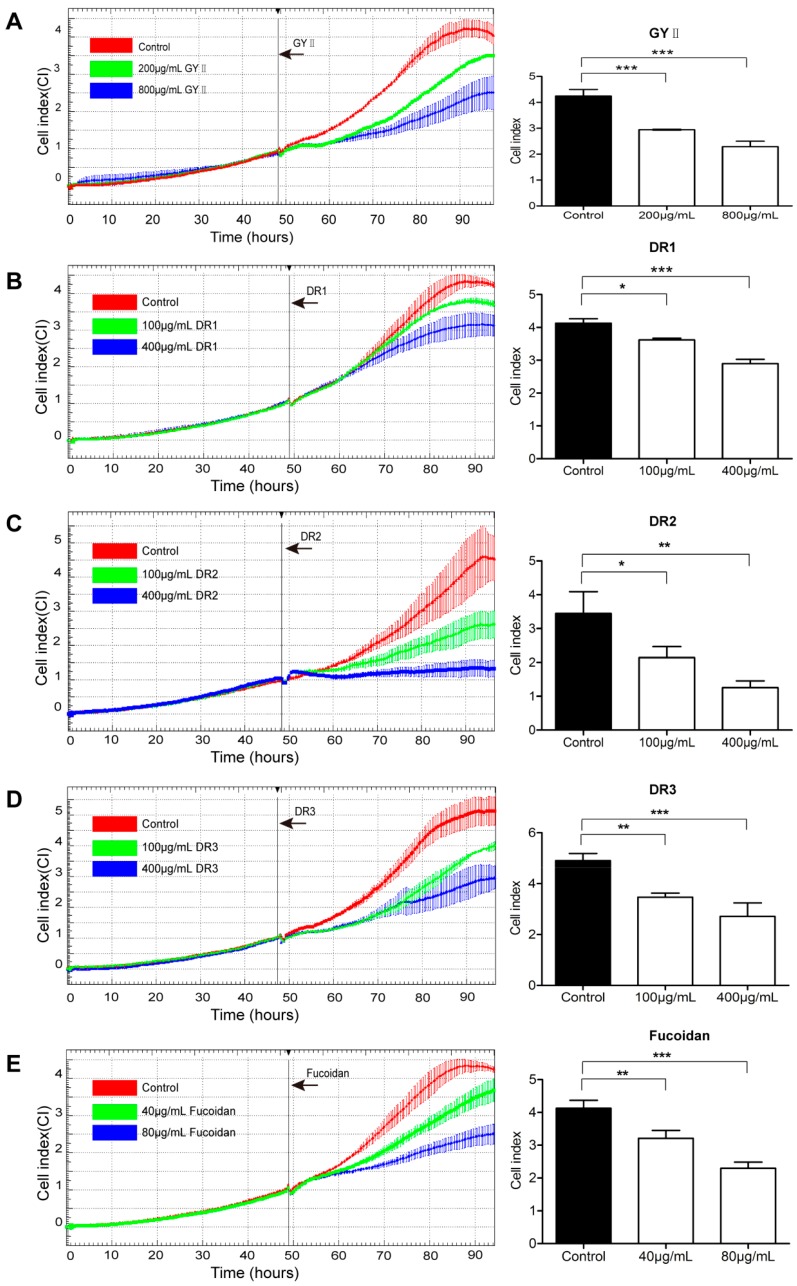
GYII and its decomposed recipes inhibited murine 4T1 cell proliferation. Time-dependent proliferation and cytotoxicity profiles of 4T1 cells were detected by real-time cell analysis (RTCA). GYII (**A**); DR1 (**B**); DR2 (**C**); DR3 (**D**); and fucoidan (**E**) diluted in medium were added to the cells when they reached the exponential growth phase (48 h after seeding). The cell index was monitored for 96 h. The cell index values from three independent experiments are presented in the right panels. (* *p* < 0.05, ** *p* < 0.01, *** *p* < 0.0001).

To identify the most effective components, the GYII formula of 13 herbs was disassembled into three decomposed recipes (DR1, DR2, and DR3) according to the TCM theory (Table 1). The inhibitory effects of GYII and its three decomposed recipes on 4T1 cells were compared. Real-time cell analysis (RTCA) was performed to determine cell numbers; an increased cell index indicated cell proliferation, while a decreased cell index indicated poor cell growth. The cellular impedance in the medium was recorded for 48 h, after which the cells were exposed to GYII or its decomposed recipes for an additional 48 h (at which point the cellular impedance of the control cells plateaued (Figure 4); red curve). Consistent with the MTT assay results, the cells exposed to GYII displayed a low cell index in comparison with that of the control cells (red curve), and this effect was dose-dependent (Figure 4A). All of the decomposed recipes had effects similar to those of GYII, even at lower concentrations (Figure 4B–D). Fucoidan was used as a positive control (Figure 4E). The cell index values at 84 h (36 h after exposure to the test compounds) are presented in the right panels of Figure 4. GYII had an inhibition rate of 46% at a concentration of 800 μg/mL, while DR2 and DR3 reached similar inhibition rates (64% and 45%, respectively) at 400 μg/mL. Fucoidan had a similar inhibition rate (44%) at a concentration of 80 μg/mL. These data indicated that GYII and its decomposed recipes had significant anti-tumor effects on 4T1 cells.

**Figure 5:**
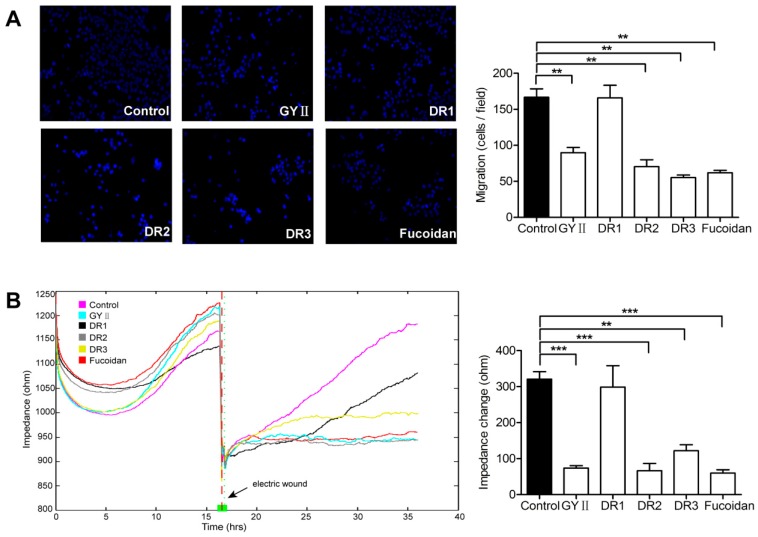
GYII and its decomposed recipes attenuated 4T1 cell migration and wound healing. (**A**) Staining of the Transwell^®^ membrane with Hoechst 33342 and visualization under a fluorescence microscope (200× magnification). The data presented in the right panel were obtained from three independent experiments by counting five randomly selected fields; (**B**) After seeding the cells, electric cell substrate impedance sensing (ECIS) was applied at 16 h to produce a lesion via electroporation (indicated by the arrows). After washing away the dead cells, fresh medium containing the test compounds were added, and the cell impedance was monitored for 20 h. Impedance changes in 20 h are presented in the right panel as the mean of three experiments (** *p* < 0.01, *** *p* < 0.0001).

Next, we used the Transwell^®^ assay to explore whether GYII and its decomposed recipes affected the migratory ability of 4T1 cells. The Transwell^®^ assay showed that all compounds except DR1 inhibited 4T1 cell trans-migration through the membrane (Figure 5A, left panel). The cells treated with GYII, DR2, and DR3 showed migration rates of 54%, 42%, and 33%, respectively, in comparison with the control cells; while DR1 did not affect migration, and fucoidan had a comparable effect to those of GYII, DR2, and DR3 (Figure 5A, right panel).

Furthermore, a wound-healing assay was conducted, in which the change in impedance was measured by electric cell substrate impedance sensing (ECIS). After electrical wounding, the lesion in the control cells (Figure 5B, pink curve) was rapidly filled by the inward migration of healthy neighboring cells to replace the dead cells, indicating the good migration ability of untreated 4T1 cells. The lesion in the cells treated with DR1 (Figure 5B, black curve) showed similar rapid filling, although the process was slower than that of the control cells. In comparison, the cells treated with GYII (Figure 5B, sky curve), DR2 (Figure 5B, grey curve), DR3 (Figure 5B, orange curve), or fucoidan (Figure 5B, red curve) essentially failed to migrate into the lesion area. These results were in agreement with the results obtained using the Transwell^®^ assay. The changes of impedance within 20 h are presented in the right panel of Figure 5B. Together, the results depicted in Figure 5 showed that GYII, DR2, and DR3 reduced the migratory activity of 4T1 cells.

### 2.4. Molecular Mechanisms of the Inhibitory Effects of GYII on Anti-Tumor Activity

**Figure 6:**
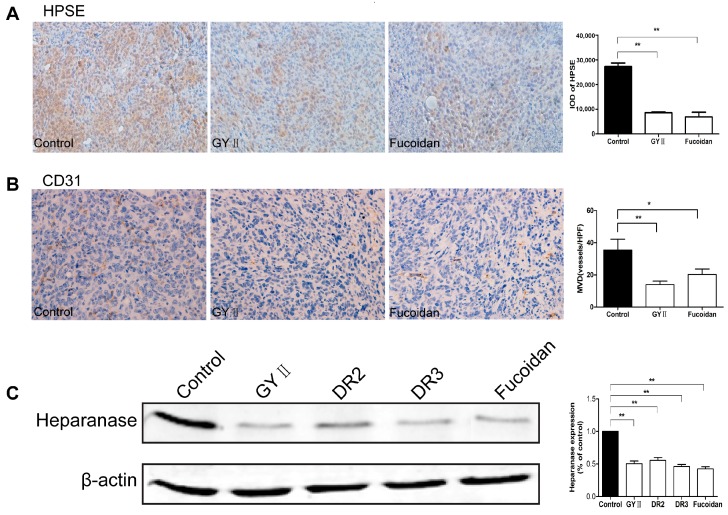
GYII and its decomposed recipes decreased the expression of heparanase and reduced angiogenesis. Formalin-fixed, paraffin-embedded 5 micron sections of the 4T1 breast tumors dissected from animals were immunostained with anti-heparanase (**A**) and anti-CD31 (**B**) antibodies (200× magnification). After exposure to the test compounds for 36 h, the total protein from each cell lysate was analyzed by Western blotting to measure the expression of heparanase (**C**). Data are presented as the mean ± SD and normalized to β-actin (* *p* < 0.05, ** *p* < 0.01).

**Figure 7:**
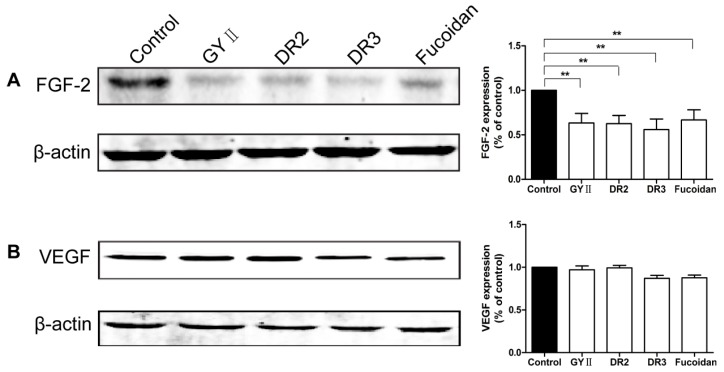
GYII and its decomposed recipes suppressed the expression of growth factors in 4T1 cells. Cells were treated with the test compounds and the expression of basic fibroblast growth factor (FGF-2) (**A**) and vascular endothelial growth factor (VEGF) (**B**) were measured by Western blotting. Data are presented as the mean ± SD and normalized to β-actin (** *p* < 0.01).

To explore the potential mechanisms underlying the effect, we examined the expression of heparanase that has been found to be correlated to tumor growth and metastasis [20]. Immunohistostaining of the tumor sections with anti-heparanase antibody revealed strong signals in the non-treated tumor sections; while the heparanase positive signals were significantly reduced in the tumor sections treated with GYII and fucoidan (Figure 6A). Furthermore, the micro-vessel density (MVD) of the tumor tissues, measured by cluster of differentiation 31 (CD31) was also reduced after GYII and fucoidan treatment (Figure 6B). In vitro, heparanase expression was also significantly reduced in cells treated with GYII, DR2, DR3, or fucoidan by 49%, 45%, 54%, and 57%, respectively, in comparison with that of the untreated control cells (Figure 6C). Moreover, it was demonstrated that GYII, DR2, DR3, or fucoidan also affected basic fibroblast growth factor (FGF-2) and vascular endothelial growth factor (VEGF) expression in 4T1 cells (Figure 7).

### 2.5. Effects of GYII and Its Decomposed Recipes on the ERK and PI3K/AKT Signaling Pathways

**Figure 8:**
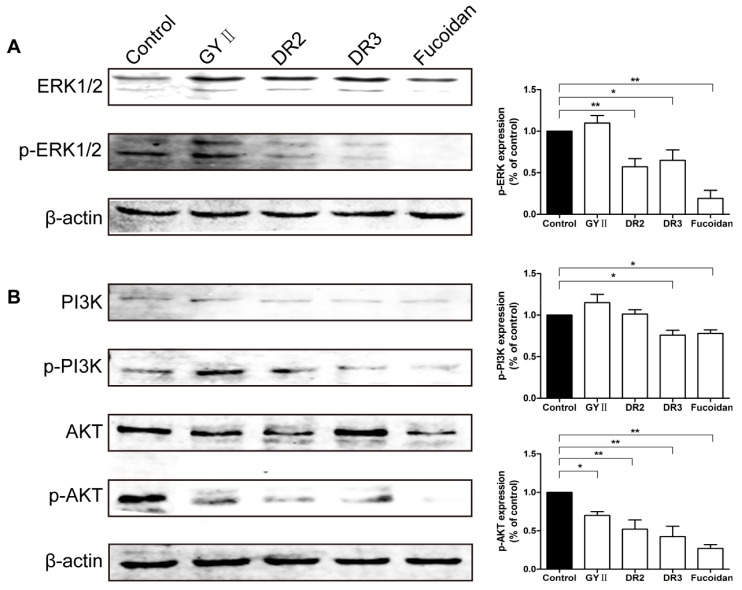
GYII and its decomposed recipes regulated the extracellular signal-regulated kinase (ERK) and phosphatidylinositol-3-kinase/ serine-threonine kinase (PI3K/AKT) pathways in 4T1 cells. Cells were treated with the test compounds and the levels of (**A**) p-ERK; (**B**) p-PI3K and p-AKT were detected by Western blotting. Data are presented as the mean ± SD and normalized to β-actin (* *p* < 0.05, ** *p* < 0.01).

The ERK and PI3K/AKT pathways play important roles in cell proliferation and migration, while heparanase expression has been found to be associated with enhanced ERK and AKT phosphorylation [21,22]. Western blot analysis revealed that DR3 and fucoidan significantly down-regulated the levels of phospho-ERK, phospho-PI3K, and phospho-AKT in comparison with the untreated control cells. In comparison, DR2 only showed a suppression effect on the phosphorylation of ERK and AKT, not PI3K. GYII decreased the levels of phospho-AKT, but with no effect on the expression of phospho-ERK and phospho-PI3K (Figure 8).

## 3. Discussion

Traditional Chinese medicine has contributed tremendously to medical care in China for millennia; however, the underlying molecular mechanisms of herbal TCM formulae and their effective components are far from being understood. Like all TCM formulae, GYII, a formula developed at the Oncology Department of the Beijing Hospital of TCM over a period of more than 30 years of clinical practice, has been demonstrated to have synergetic effects with chemotherapy [4,8]. Experimental studies have reported that GYII inhibits tumor growth by inducing apoptosis or autophagy in lung or breast cancer xenografts [9,10]. However, the anti-tumor effects and the mechanisms of the action remain to be fully elucidated. 

In the present study, we found that GYII not only inhibited the growth of primary tumors but also possessed anti-metastatic activity in a breast cancer model. The same potency of GYII was also evidenced in tumor cell models. These data provided an evidence for the anti-metastatic efficacy of GYII. In addition, GYII displayed differential inhibitory effects on cell proliferation, with significant inhibitory activity on 4T1 and MCF-7 cells, but a marginal effect on MDA-MB-231 cells. The distinct response of 4T1 and MCF-7 cells to GYII may suggest that specific molecules targeted by GYII are profoundly expressed in these cells. 

Though the herbal medicine formula has been applied to patients, a clear drawback of the classical formula is their complicated composition, which largely prevented the development of these herbal medicines. One important finding of this study is that the decomposed recipes except DR1 showed stronger inhibitory effects on cell proliferation and migration at a lower dose compared to GYII. GYII had an inhibition rate of 46% at the concentration of 800 μg/mL, while DR2 and DR3 reached similar inhibition rates (64% and 45%, respectively) at 400 μg/mL. Moreover, the cells treated with GYII, DR2, and DR3 showed migration rates of 54%, 42%, and 33%, respectively, compared to the control group, while DR1 had essentially no activity. This result indicates that the six herbs constituting DR2 and DR3 (three each) out of the 13 herbs in GYII are likely to contain higher bioactive anti-cancer components. Furthermore, the DR3 treatment resulted in a 54% decrease of heparanase expression, while DR2 had a 45% decrease, indicating a differential effect between the components in DR2 and DR3. Interestingly, this finding is in agreement with classical TCM theory; DR1 is composed of herbs mainly associated with the replenishment (*yiqi*) effect that may primarily affect the immune system instead of direct cytotoxicity [23,24]. In contrast, the herbs included in DR2 and DR3 are characterized by the stimulation of blood circulation and neutralizing tumor-induced toxicity, commonly used for removing lumps or protuberances [25]. Current studies have also demonstrated that herbs with *huoxuehuayu* and *jiedusanjie* functions are able to kill tumor cells directly and attenuate tumor growth and metastasis [26,27]. Thus, our results suggest that the herbs in the DR2 and DR3 may contain anti-cancer and anti-metastatic components that can be further investigated. Extensive studies have confirmed that heparanase, which participates in the degradation of the ECM, is overproduced in several types of tumor including breast cancer [20,28,29]. This kind of characteristic is often associated with increased aggressiveness, higher microvessel density, and poorer clinical prognosis [21,30,31]. Heparanase overexpression enhances the phosphorylation of molecules in the ERK and AKT pathways to stimulate the proliferation and migration of tumor cells [22,32]. At the same time, heparanase cleaves heparan sulfate and releases growth factors such as FGF and VEGF from ECM storage, also leading to the ERK and AKT pathways [20,21,33]. Accumulated evidence indicates that the drugs targeting heparanase reduce angiogenesis, inhibit spontaneous metastasis, and prolong survival in cancer models [34,35,36,37].

In the present study, GYII attenuated heparanase expression and angiogenesis in murine primary breast tumors. Moreover, GYII and its decomposed recipes treatment decreased the expression of heparanase and growth factors in 4T1 cells, which was partly related to the phosphorylation of ERK and AKT. These findings suggest a potential mechanism of GYII’s anti-tumor and anti-metastatic effect. There are some natural substances, including arctigenin [38], matrine [39], and NAX014 (a synthetic derivative 13-(4-chlorophenylethyl) berberine iodide) [40] that decrease heparanase expression, which are partly responsible for its anti-tumor and anti-metastatic activity. The effective dosage of the natural substances is less than that of GYII, which indicates the necessity to explore the more active components of GYII. The study of NAX014 implied that NAX014-dependent inhibition of heparanase could determine the lower vessel density and the reduced tumor vascularization could also be related to the decreased intratumoral immune infiltration, which may affect the anti-tumor activity. As for the effect of GYII on immune infiltration after the heparanase decrease, it still deserves further investigation. Furthermore, the results from Figure 8 showed that GYII and its decomposed recipes differently regulated the ERK and PI3K/AKT pathways. This indicates that GYII and its decomposed recipes may simultaneously target multiple molecules or pathways and exhibit distinct regulative effects on pathways due to multiple active chemical components of formulae.

## 4. Materials and Methods

### 4.1. Chemicals and Reagents

Fucoidan was purchased from Sigma-Aldrich (St. Louis, MO, USA). The primary antibodies against heparanase (ab85543), VEGF (ab46154), and CD31 (ab28364) were purchased from Abcam Biotechnology (Cambridge, UK). The primary antibodies against FGF-2 (sc-79) and beta-actin (sc-47778) were purchased from Santa Cruz Biotechnology (Santa Cruz, CA, USA). The primary antibodies against ERK (#9102), phospho-ERK (#4377), PI3K (#4292), phospho-PI3K (#4228), AKT (#9272), and phospho-AKT (#4058) were purchased from Cell Signaling Technology (Beverly, MA, USA). Dylight 800-conjugated goat anti-mouse secondary antibodies (072-07-18-06) and Dylight 680-conjugated goat anti-rabbit IgG secondary antibodies (072-06-15-06) were purchased from KPL, Inc. (Gaithersburg, MD, USA).

### 4.2. Preparation of GYII and Its Decomposed Recipes

GYII is composed of 13 species of herbs used in TCM. The compositions of GYII and its decomposed recipes are listed in Table 1. The compounds used in the study were manufactured under good manufacturing practice (GMP) regulations at Capital Medical University following the protocol described in the Chinese Pharmacopoeia 2010 [41]. To prepare the extracts, the dry herbs were mixed in the ratios shown in Table 1, and the extracts of GYII and the decomposed recipes were prepared as previously described with slight modifications [42,43]. Briefly, (1) defatting with petroleum ether; (2) water extracting-alcohol precipitating; (3) deproteinization. This 3-step process yielded 1 g of GYII from 16 g of herbs, 1 g of DR1 from 11 g of herbs, 1 g of DR2 from 23 g of herbs, and 1 g of DR3 from 16 g of herbs. All of the compounds were dissolved in 0.9% NaCl for in vivo experiments or in cell culture medium for in vitro experiments, followed by filtration through a 0.22 µm Millex syringe filter (EMD Millipore, Billerica, MA, USA) under sterile conditions. 

### 4.3. Cell Culture

The 4T1 murine breast cancer cell line (Cell Bank of the Chinese Academy of Sciences, Shanghai, China) and the 4T1-luc2 cell line (high luciferase expression and high metastatic rate, acquired from Caliper Life Sciences, Alameda, CA, USA) were cultured in RPMI-1640 medium (Gibco^®^, Life Technologies, Carlsbad, CA, USA) supplemented with 10% fetal bovine serum (FBS) (Gibco^®^) and 1% penicillin-streptomycin (Hyclone, Logan, UT, USA). The MCF-7 and MDA-MB-231 human breast cancer cell lines (Cell Center of the Medical Research Institute of the Chinese Academy of Medical Sciences, Beijing, China) were cultured in Dulbecco’s modified Eagle’s medium (DMEM) (Gibco^®^) or RMPI-1640 medium with 10% FBS and 1% penicillin-streptomycin, respectively. All cells were cultured at 37 °C in a humidified incubator (Sanyo, Osaka, Japan) with a 5% CO_2_ atmosphere.

### 4.4. Murine 4T1-luc2 Breast Carcinoma Model

The tumor cell implantation experiments and animal care procedures were approved by the Institutional Animal Use and Care Committee of Capital Medical University (No. AEEI-2014-052) and were conducted in accordance with the Provision and General Recommendation of Chinese Experimental Animals Administration Legislation. Female balb/c mice (8–10 weeks old) were obtained from Vital River Laboratory Animal Technology Co. Ltd. (Beijing, China) for use in all in vivo experiments. All surgeries were performed in animals anesthetized with pentobarbital (60 mg/kg i.p.) and inhaled isoflurane, and efforts were made to minimize animal suffering.

Orthotopic implantation of 4T1-luc2 cells into the mammary fat pads of balb/c mice was performed as described previously [44]. Briefly, female balb/c mice (8–10 weeks old) were orthotopically inoculated with 4T1-luc2 mammary carcinoma cells (1 × 10^4^) in the mammary fat pads under anesthesia. Tumor length (L) and width (W) were measured twice weekly with a caliper, and the tumor volume (V) was calculated (V = (L × W^2^)/2). Treatment was commenced 7 days after implantation, when the tumor volume reached 30–50 mm^3^. The mice were divided into 3 groups (n = 6/group) with similar tumor size distributions. The animals were treated daily with the extract of GYII (5.0 g/kg), fucoidan (0.2 g/kg), or vehicle (0.9% NaCl) by intragastric administration. 

Moreover, the bioluminescence images of the GYII and control group were monitored. The mice were injected intraperitoneally with D-luciferin at 150 mg/kg (Gold Biotechnology, St. Louis, MO, USA), and then bioluminescence images were taken using IVIS Spectrum. Light outputs were quantified using Living Image 4.3.1 (Caliper Life Sciences, Alameda, CA, USA). The experiment was terminated after 25 days of treatment (32 days after tumor cell implantation) and the primary tumors were cut off for weighting and further analysis. After primary tumor resection, animals were detected for lung metastasis with bioluminescent imaging. The anti-tumor activity was expressed as the tumor weight inhibitory rate (IR). 

### 4.5. MTT Assay

Cell viability was measured using the 3-(4,5-dimethylthiazol-2-yl)-2,5-diphenyltetrazolium bromide (MTT) assay. The cells were seeded into 96-well plates (Corning, Corning, NY, USA) at a density of 2 × 10^3^ cells/well (6.25 × 10^3^ cells/cm^2^), incubated overnight at 37 °C with 5% CO_2_, and exposed to various concentrations of GYII (200–800 μg/mL) for 24 or 48 h. After adding 15 μL of 5 mg/mL MTT (Sigma-Aldrich, St. Louis, MO, USA) solution, the cells were cultured for 4 h. After removal of the solution, the cells were lysed in 150 μL DMSO and absorbance was measured at 570 nm using a microplate reader (Thermo Scientific, Waltham, MA, USA). The optical density (OD) was used to calculate the cell viability after treatment with different concentrations of GYII. 

$$\mathrm{Cell}\;\mathrm{viability}=\frac{\left(\mathrm{OD}\;\mathrm{of}\;\mathrm{experimental}\;\mathrm{sample}-\mathrm{OD}\;\mathrm{of}\;\mathrm{blank}\;\mathrm{group}\right)}{\left(\mathrm{OD}\;\mathrm{of}\;\mathrm{control}\;\mathrm{group}-\;\mathrm{OD}\;\mathrm{of}\;\mathrm{blank}\;\mathrm{group}\right)}\times 100\text{\%}$$

### 4.6. RTCA for Cell Proliferation

Real-time cell analysis (RTCA) of proliferation was performed using a xCELLigence DP instrument (Roche Applied Science, Mannheim, Germany), which measures the changes in electrical impedance when the cells attached and covered approximately 80% of the area on the bottom of a culture dish covered with a gold microelectrode array. The electrical impedance corresponded to the cellular abundance, implying cell proliferation. The experiments were performed following the manufacturer’s instruction and reference [45]. Briefly, 2.5 × 10^3^ cells/well (100 μL) was added to each well of an E-plate 16, which was left at room temperature for 30 min to allow the cells to settle evenly onto the bottom of each well. The plate was placed on the RTCA DP analyzer and cell growth was recorded in an incubator at 37 °C with 5% CO_2_. When the cells reached the exponential growth phase (about 48 h, cell index near 1), the old medium was replaced with fresh medium containing the test compounds (200 μg/mL or 800 μg/mL GYII; 100 μg/mL or 400 μg/mL DR1, DR2, or DR3; 40 μg/mL or 80 μg/mL fucoidan), and the xCELLigence system recorded cell proliferation every 15 min.

### 4.7. Transwell^®^ Migration Assay

First, the cells were treated with the test compounds (450 μg/mL GYII; 200 μg/mL DR1, DR2, or DR3; or 40 μg/mL fucoidan) in culture flasks for 36 h. Next, 5 × 10^4^ cells/well in 100 μL serum-free medium were transferred into the upper chamber of a Transwell^®^ device (Corning, Corning, NY, USA) and were allowed to migrate towards the bottom chamber, which contained 600 μL of RPMI-1640 medium with 10% FBS. After 24 h of migration, cells on the top side of the membrane were removed with a cotton swab, and the cells remaining on the bottom side of the membrane were fixed using methanol for 10 min at −20 °C and stained with Hoechst 33342 (Sigma-Aldrich, St. Louis, MO, USA) for 15 min at 25 °C. After 3 washes with PBS, the cells were counted under a fluorescence microscope at 200× magnification (Leica, Heidelberg, Germany). The average number of cells in 5 randomly chosen fields was reported.

### 4.8. ECIS Wound Healing Assay

Electric cell substrate impedance sensing (ECIS) was used to measure the wound-healing capacity of cells [46]. A suspension of 4T1 cells was prepared (2 × 10^5^ cells/mL), and 400 μL of the cell suspension was added to each well of a 8W10E+ ECIS plate (Applied Biophysics, Troy, NY, USA). When the cells grew to confluence (around 16 h), the cell layer was electrically wounded by contact with a 250 µm (diameter) electrode (current: 6500 μA; frequency: 100,000 Hz; time: 60 s). After the lesion was formed, dead cells were washed away and fresh basal media containing the test compounds (450 μg/mL GYII; 200 μg/mL DR1, DR2, or DR3; or 40 μg/mL fucoidan) were added. Wound healing was assessed by continuous impedance measurements for 20 h.

### 4.9. Western Blotting

After exposure to the test compounds (450 μg/mL GYII; 200 μg/mL DR2 and DR3; or 40 μg/mL fucoidan) for 36 h, the 4T1 cells were harvested and lysed with RIPA lysis buffer (Beyotime Biotechnology, Beijing, China) containing Protease Inhibitor Cocktail Set III (Calbiochem, San Diego, CA, USA). The cell lysates were centrifuged at 12,000 rpm for 15 min at 4 °C, and the supernatants were collected. The protein concentration was determined using the Pierce BCA Assay Kit (Thermo Scientific, Rockford, IL, USA). Equal amounts (45 μg/lane) of total protein were separated by 12% SDS-PAGE and transferred onto PVDF membranes. The membranes were blocked with 5% BSA (Amresco, Solon, OH, USA) at room temperature for 1 h and incubated overnight at 4 °C with the following primary antibodies: anti-heparanase (1:500), anti-FGF-2 (1:500), anti-VEGF (1:1000), anti-ERK/p-ERK (1:1000), anti-PI3K/p-PI3K (1:1000), anti-AKT/p-AKT (1:1000), and β-actin (1:1000). After washing the membranes in Tris-buffered saline with 0.1% Tween-20 (TBST), the membranes were probed with secondary antibodies (1:10,000) for 1 h at room temperature. The signals were detected using an Odyssey Infrared Imaging System (Li-cor Biosciences, Lincoln, NE, USA). The relative density of the protein bands was measured by Odyssey version 3.0 software (LI-COR Biosciences). The experiments were repeated three times.

### 4.10. Immunohistochemistry

Paraffin-embedded mouse tumor sections were deparaffinized, rehydrated, and endogenous peroxidase activity was quenched (30 min) by 3% hydrogen peroxide in methanol. Following two washes in PBS, antigen retrieval was performed by boiling (20 min) in 10 mM citrate buffer. Sections were blocked with 10% normal goat serum in PBS for 60 min and incubated (overnight, 4 °C) with anti-heparanase polyclonal antibody diluted 1:100 and anti-CD31 polyclonal antibody diluted 1:100. Sections were washed twice in PBS and incubated with polyperoxidase-anti-rabbit IgG (ZSGB-BIO Inc., Co., Ltd., Beijing, China) at 37 °C for 30 min. Following additional washes, the color was developed with diaminobenzidine buffer (DAB), and sections were counterstained with hematoxylin. The sections were viewed under a microscope (Nikon Eclipse TS100, Tokyo, Japan) with 200× magnification. Five randomly selected fields of each section were analyzed by Image-Pro Plus 6.0 image analysis software. The integral optical density (IOD) was calculated. Micro-vessel density (MVD) was measured in five fields with a higher density of CD31-positive cells and cell clusters. The presence of visible blood vessel lumen was not required to be defined as positive [47].

### 4.11. Statistical Analysis

Statistical significance was determined by one-way ANOVA or *t*-test. All calculations were performed using GraphPad Prism version 5.0 (GraphPad Software Inc., San Diego, CA, USA). The significance threshold was *p* < 0.05.

## 5. Conclusions

GYII exerts anti-tumor growth and anti-metastatic effects on a murine breast cancer model. The anti-tumor activity of GYII and its decomposed recipes is, at least partly, associated with decreased heparanase and growth factor expression, which subsequently suppressed the activation of the ERK and AKT pathways, given that HS in ECM serves as a substrate of heparanase and acts in concert with growth factors such as FGF-2 and VEGF to promote tumor growth and metastasis. Further investigations regarding HS expression and structural alterations associated with heparanase expression in the cells are required to elucidate the molecular mechanisms of GYII and its decomposed recipes in the future.

## Figures and Tables

**Table 1:** Herbal composition of GYII and its decomposed recipes.

Whole Prescription	Decomposed Recipes	Components	Ratio in GYII (%)	Ratio in Decomposed Recipes (%)
**GYII**	**DR1**	*Codonopsis pilosula* (Franch.) Nannf.	7.6	14.3
		*Poria cocos* (Schw.). Wolf.	5.1	9.5
		*Atractylodis macrocephala* Koidz.	5.1	9.5
		*Astragalus membranaceus* (Fisch.) Bge. var. *mongholicus* (Bge.) Hsiao	15.2	28.6
		*Ligustrum lucidum* Ait.	7.6	14.3
		*Lycium barbarum* L.	7.6	14.3
		*Epimedium brevicornum* Maxim	5.1	9.5
	**DR2**	*Ligusticum chuanxiong* Hort.	5.1	20.0
		*Spatholobus suberectus* Dunn	15.2	60.0
		*Curcuma kwangsiensis* S. G. Lee et C. F. Liang	5.1	20.0
	**DR3**	*Sarcandra glabra* (Thunb.) Nakai	7.6	35.7
		*Fritillaria thunbergii* Miq.	7.6	35.7
		*Sophora flavescens* Ait.	6.1	28.6

NOTES: GYII = *Gubenyiliu* II formula; DR1 = decomposed recipe 1; DR2 = decomposed recipe 2; DR3 = decomposed recipe 3.

## Abbreviations

**Abbreviation**	**Full Name**
AKT	Serine-threonine kinase
BAP	Blood activation prescription
CD31	Cluster of differentiation 31
CEA	Carcino-embryonic antigen
DMSO	Dimethyl sulphoxide
ECIS	Electric cell substrate impedance sensing
ECM	Extracellular matrix
ERK	Extracellular signal-regulated kinase
FGF-2	Basic fibroblast growth factor
FBS	Fetal bovine serum
HS	Heparan sulfate
HPSE	Heparanase
IC50	Half-maximal inhibitory concentrations
IHC	Immunohistochemistry
IR	Inhibitory rate
IVIS	In-vivo imaging system
MTT	3-(4,5-dimethyl-thiazol-2-yl)-2,5-Diphenyl tetrazolium bromide
MVD	Micro-vessel density
OD	Optical density
PI3K	Phosphatidylinositol-3-kinase
PBS	Phosphate buffer solution
RTCA	Real-time cell analysis
SDS-PAGE	Sodium dodecyl sulfate-polyacrylamide gel electrophoresis
TCGA	The cancer genome atlas
VEGF	Vascular endothelial growth factor

## References

[1] Torre L.A., Bray F., Siegel R.L., Ferlay J., Lortet-Tieulent J., Jemal A. (2015). Global cancer statistics, 2012. CA Cancer J. Clin..

[2] Li X., Yang G., Li X., Zhang Y., Yang J., Chang J., Sun X., Zhou X., Guo Y., Xu Y. (2013). Traditional Chinese medicine in cancer care: A review of controlled clinical studies published in Chinese. PLoS ONE.

[3] Qi F., Zhao L., Zhou A., Zhang B., Li A., Wang Z., Han J. (2015). The advantages of using traditional Chinese medicine as an adjunctive therapy in the whole course of cancer treatment instead of only terminal stage of cancer. Biosci. Trends.

[4] Zhang Q., Yu R., Tang W., Wang X., Rao X., Wang Y., Guan T., Zhao W. (2000). The clinical research of *Gubenyiliu* II on tumors. Chin. J. Inf. TCM.

[5] Li S.G., Chen H.Y., Ou-Yang C.S., Wang X.X., Yang Z.J., Tong Y., Cho W.C. (2013). The efficacy of Chinese herbal medicine as an adjunctive therapy for advanced non-small cell lung cancer: A systematic review and meta-analysis. PLoS ONE.

[6] Xiong F., Jiang M., Huang Z., Chen M., Chen K., Zhou J., Yin L., Tang Y., Wang M., Ye L. (2014). A novel herbal formula induces cell cycle arrest and apoptosis in association with suppressing the PI3K/AKT pathway in human lung cancer A549 cells. Integr. Cancer Ther..

[7] Liu J., Yu R.C., Tang W.J. (2002). Influence of combined therapy of guben yiliu III, moxibustion and chemotherapy on immune function and blood coagulation mechanism in patients with mid-late stage malignant tumor. Zhongguo Zhong Xi Yi Jie He Za Zhi.

[8] Yang G., Xu Y., Fu Q., Han D., Yu J., Yang Z., Tang W. (2008). Clinical Observation on Guben Yiliu II Combined with Chemotherapy in the Treatment of 28 Cases of Advanced Breast Cancer. J. Tradit. Chin. Med..

[9] Zhang G., Wang X., Li P., Yang G., Tang Y., Liu X., Sheng X. (2007). Inhibitory effect of *Gubenyiliu* Formula II and its combinative effect with chemotherapeutants on mouse Lewis lung carcinoma. J. Beijing Univ. Tradit. Chin. Med..

[10] Ma C., Wang X.M., Yu M.W., Zhang G.L., Nan N., Zhang Y., Cao K.X., Li J.P. (2014). Inhibitory effects of Guben Yiliu Formula II (II) and its blood activation prescriptions on the growth of MCF-7 human breast cancer xenografts in nude mice. Chin. J. Integr. Med..

[11] Langley R.R., Fidler I.J. (2011). The seed and soil hypothesis revisited—The role of tumor-stroma interactions in metastasis to different organs. Int. J. Cancer.

[12] Deng W., Sui H., Wang Q., He N., Duan C., Han L., Li Q., Lu M., Lv S. (2013). A Chinese herbal formula, Yi-Qi-Fu-Sheng, inhibits migration/invasion of colorectal cancer by down-regulating MMP-2/9 via inhibiting the activation of ERK/MAPK signaling pathways. BMC Complement. Altern. Med..

[13] Wang N., Feng Y., Cheung F., Wang X., Zhang Z., Feng Y. (2015). A Chinese medicine formula Gegen Qinlian decoction suppresses expansion of human renal carcinoma with inhibition of matrix metalloproteinase-2. Integr. Cancer Ther..

[14] Lin W., Zhuang Q., Zheng L., Cao Z., Shen A., Li Q., Fu C., Feng J., Peng J. (2015). Pien Tze Huang inhibits liver metastasis by targeting TGF-beta signaling in an orthotopic model of colorectal cancer. Oncol. Rep..

[15] Vlodavsky I., Friedmann Y., Elkin M., Aingorn H., Atzmon R., Ishai-Michaeli R., Bitan M., Pappo O., Peretz T., Michal I. (1999). Mammalian heparanase: Gene cloning, expression and function in tumor progression and metastasis. Nat. Med..

[16] Li J.P. (2008). Heparin, heparan sulfate and heparanase in cancer: Remedy for metastasis?. Anticancer Agents Med. Chem..

[17] Sun X., Zhang G., Nian J., Yu M., Chen S., Zhang Y., Yang G., Yang L., Cheng P., Yan C. (2017). Elevated heparanase expression is associated with poor prognosis in breast cancer: a study based on systematic review and TCGA data. Oncotarget.

[18] Kwak J.Y. (2014). Fucoidan as a marine anticancer agent in preclinical development. Mar. Drugs.

[19] Xue M., Ge Y., Zhang J., Wang Q., Hou L., Liu Y., Sun L., Li Q. (2012). Anticancer properties and mechanisms of fucoidan on mouse breast cancer in vitro and in vivo. PLoS ONE.

[20] Cohen I., Pappo O., Elkin M., San T., Bar-Shavit R., Hazan R., Peretz T., Vlodavsky I., Abramovitch R. (2006). Heparanase promotes growth, angiogenesis and survival of primary breast tumors. Int. J. Cancer.

[21] Ilan N., Elkin M., Vlodavsky I. (2006). Regulation, function and clinical significance of heparanase in cancer metastasis and angiogenesis. Int. J. Biochem. Cell Biol..

[22] Vlodavsky I., Elkin M., Ilan N. (2011). Impact of heparanase and the tumor microenvironment on cancer metastasis and angiogenesis: Basic aspects and clinical applications. Rambam Maimonides Med. J..

[23] Sultan M.T., Butt M.S., Qayyum M.M., Suleria H.A. (2014). Immunity: Plants as effective mediators. Crit. Rev. Food Sci. Nutr..

[24] Guo Q., Li J., Lin H. (2015). Effect and Molecular Mechanisms of Traditional Chinese Medicine on Regulating Tumor Immunosuppressive Microenvironment. BioMed Res. Int..

[25] Chen T., Li D., Fu Y.L., Hu W. (2008). Screening of QHF formula for effective ingredients from Chinese herbs and its anti-hepatic cell cancer effect in combination with chemotherapy. Chin. Med. J..

[26] Liang F., Li L., Wang M., Niu X., Zhan J., He X., Yu C., Jiang M., Lu A. (2013). Molecular network and chemical fragment-based characteristics of medicinal herbs with cold and hot properties from Chinese medicine. J. Ethnopharmacol..

[27] Yin J.H., Shi W.D., Zhu X.Y., Chen Z., Liu L.M. (2012). Qingyihuaji formula inhibits progress of liver metastases from advanced pancreatic cancer xenograft by targeting to decrease expression of Cyr61 and VEGF. Integr. Cancer Ther..

[28] El-Assal O.N., Yamanoi A., Ono T., Kohno H., Nagasue N. (2001). The clinicopathological significance of heparanase and basic fibroblast growth factor expressions in hepatocellular carcinoma. Clin. Cancer Res..

[29] Doviner V., Maly B., Kaplan V., Gingis-Velitski S., Ilan N., Vlodavsky I., Sherman Y. (2006). Spatial and temporal heparanase expression in colon mucosa throughout the adenoma-carcinoma sequence. Mod. Pathol..

[30] Vlodavsky I., Friedmann Y. (2001). Molecular properties and involvement of heparanase in cancer metastasis and angiogenesis. J. Clin. Investig..

[31] Tang D., Zhang Q., Zhao S., Wang J., Lu K., Song Y., Zhao L., Kang X., Wang J., Xu S. (2013). The expression and clinical significance of microRNA-1258 and heparanase in human breast cancer. Clin. Biochem..

[32] Yuan L., Hu J., Luo Y., Liu Q., Li T., Parish C.R., Freeman C., Zhu X., Ma W., Hu X. (2012). Upregulation of heparanase in high-glucose-treated endothelial cells promotes endothelial cell migration and proliferation and correlates with Akt and extracellular-signal-regulated kinase phosphorylation. Mol. Vis..

[33] Suzuki T., Yasuda H., Funaishi K., Arai D., Ishioka K., Ohgino K., Tani T., Hamamoto J., Ohashi A., Naoki K. (2015). Multiple roles of extracellular fibroblast growth factors in lung cancer cells. Int. J. Oncol..

[34] Hammond E., Brandt R., Dredge K. (2012). PG545, a heparan sulfate mimetic, reduces heparanase expression in vivo, blocks spontaneous metastases and enhances overall survival in the 4T1 breast carcinoma model. PLoS ONE.

[35] Zhou H., Roy S., Cochran E., Zouaoui R., Chu C.L., Duffner J., Zhao G., Smith S., Galcheva-Gargova Z., Karlgren J. (2011). M402, a novel heparan sulfate mimetic, targets multiple pathways implicated in tumor progression and metastasis. PLoS ONE.

[36] Ferro V., Dredge K., Liu L., Hammond E., Bytheway I., Li C., Johnstone K., Karoli T., Davis K., Copeman E. (2007). PI-88 and novel heparan sulfate mimetics inhibit angiogenesis. Semin. Thromb. Hemost..

[37] Miao H.Q., Elkin M., Aingorn E., Ishai-Michaeli R., Stein C.A., Vlodavsky I. (1999). Inhibition of heparanase activity and tumor metastasis by laminarin sulfate and synthetic phosphorothioate oligodeoxynucleotides. Int. J. Cancer.

[38] Lou C., Zhu Z., Zhao Y., Zhu R., Zhao H. (2017). Arctigenin, a lignan from Arctium lappa L., inhibits metastasis of human breast cancer cells through the downregulation of MMP-2/-9 and heparanase in MDA-MB-231 cells. Oncol. Rep..

[39] Liu X.Y., Fang H., Yang Z.G., Wang X.Y., Ruan L.M., Fang D.R., Ding Y.G., Wang Y.N., Zhang Y., Jiang X.L. (2008). Matrine inhibits invasiveness and metastasis of human malignant melanoma cell line A375 in vitro. Int. J. Dermatol..

[40] Pierpaoli E., Damiani E., Orlando F., Lucarini G., Bartozzi B., Lombardi P., Salvatore C., Geroni C., Donati A., Provinciali M. (2015). Antiangiogenic and antitumor activities of berberine derivative NAX014 compound in a transgenic murine model of HER2/neu-positive mammary carcinoma. Carcinogenesis.

[41] Chinese Pharmacopoeia Commission (2010). Pharmacopoeia of the People’s Republic of China.

[42] Zhao X.N., Liang J.L., Chen H.B., Liang Y.E., Guo H.Z., Su Z.R., Li Y.C., Zeng H.F., Zhang X.J. (2015). Anti-Fatigue and Antioxidant Activity of the Polysaccharides Isolated from Millettiae speciosae Champ. Leguminosae. Nutrients.

[43] Liu H., Zhang W., Dong S., Song L., Zhao S., Wu C., Wang X., Liu F., Xie J., Wang J. (2015). Protective effects of sea buckthorn polysaccharide extracts against LPS/d-GalN-induced acute liver failure in mice via suppressing TLR4-NF-kappaB signaling. J. Ethnopharmacol..

[44] Jenkins D.E., Oei Y., Hornig Y.S., Yu S.F., Dusich J., Purchio T., Contag P.R. (2003). Bioluminescent imaging (BLI) to improve and refine traditional murine models of tumor growth and metastasis. Clin. Exp. Metastasis.

[45] Limame R., Wouters A., Pauwels B., Fransen E., Peeters M., Lardon F., De Wever O., Pauwels P. (2012). Comparative analysis of dynamic cell viability, migration and invasion assessments by novel real-time technology and classic endpoint assays. PLoS ONE.

[46] Szulcek R., Bogaard H.J., van Nieuw A.G. (2014). Electric cell-substrate impedance sensing for the quantification of endothelial proliferation, barrier function, and motility. J. Vis. Exp..

[47] Weidner N., Semple J.P., Welch W.R., Folkman J. (1991). Tumor angiogenesis and metastasis—Correlation in invasive breast carcinoma. N. Engl. J. Med..

